# The Influence of the Common Cold on Self-Rated Health: A Population-Based Study

**DOI:** 10.3934/publichealth.2015.3.247

**Published:** 2015-06-24

**Authors:** Christy Pu

**Affiliations:** 1 Institute of Hospital and Health Care Administration, School of Medicine, National Yang-Ming University, Taipei 112, Taiwan;

**Keywords:** self-rated health, common cold, chronic conditions, Charlson comorbidity, fractional polynomial, Taiwan

## Abstract

**Objective:**

Studies on the association between self-rated health and acute conditions are sparse. The aim of this study was to examine whether individuals respond to acute conditions (such as the common cold) in health ratings as well as the effect of chronic conditions (using the Charlson comorbidity score) on self-rated health.

**Methods:**

The national representative survey data was linked with the claims data from the Taiwan National Health Insurance for 13,723 adults ≥ 18 years. Ordered logistic regressions with fractional polynomials were estimated to determine the relationship between the frequency of common cold episodes and the Charlson comorbidity score on self-rated health. The interactions between these two variables and the baseline age were tested.

**Results:**

Self-rated health worsens with the increased frequency of both common cold episodes and the Charlson comorbidity score. Both variables have a non-linear relationship with self-rated health. Younger individuals put heavier weight on acute health conditions than their older counterparts.

**Conclusion:**

Individuals respond to questions regarding their self-rated health based on their acute health condition along with chronic condition. Thus the information on self-rated health depends on the timing the information is collected, and whether at that time the individual experienced acute health conditions or not.

## 1. Introduction

Many studies have investigated how a person's self-rated health (SRH) is related to chronic diseases, but none of them examined whether individuals were influenced by the frequency of their common cold episodes when rating their health. This issue is important, because if individuals are influenced by the frequency that they contract the common cold when rating their health, then the accuracy of using a person's SRH for measuring the need of medical resources, or as a risk assessment indicator must be taken into consideration. Self-rated health has been widely investigated in health services research, measured by using the single question “in general, how would you rate your general health?” and the mutually exclusive response options of “excellent, very good, good, fair, or poor” mainly because of its use in risk adjustment. This variable gained the attention of researchers because studies showed its association with mortality [1], as well as medical service utilization [2],[3]. Due to its popularity and usefulness in risk prediction, many validation studies have investigated how SRH is associated with objective health measures. Some studies tested the association between SRH and clinical factors [4],[5], such as the assessment of a patient's health by their physician [6],[7], and other well-known risk-adjustment systems such as the Diagnostic Cost Groups (DCGs) and Adjusted Clinical Groups (ACGs) [8]. Most studies tend to agree that SRH is closely associated with these objective health measures.

An extensive amount of studies have been conducted to determine how SRH is related to chronic diseases such as chronic renal disease [9] or chronic heart failure [10]. Several studies also used self-reported condition checklists [11],[12]. These studies often find that there is a significant relationship between SRH and chronic conditions. Previous studies showed that there was a significant association between the comorbidity and SRH [11],[13]. The Charlson comorbidity index is a widely used measure of comorbidity that involves several categories of diseases with a weighted severity [14]. Although the Charlson comorbidity index was not designed to measure chronic diseases, the categories of diseases involved are either chronic or chronic in nature. For example, HIV/AIDS is often characterized as a chronic rather than an acute disease [15]. It has been shown that this index is a significant and independent predictor of patient survival [16]–[18], and is associated with treatment decisions, clinical outcomes, and resource utilization [19],[20]. Heller et al. [13] investigated the relationship between SRH and the Charlson comorbidity score by linking survey data with Medicare claims data. They found that SRH is closely associated with the Charlson Comorbidity score, and that the responsiveness of SRH to the baseline Charlson comorbidity is nonlinear.

Despite the fact that people usually recover rather quickly from the common cold, it is reasonable to hypothesize that individuals will respond to it if it happens frequently. If that is the case, then the year in which a person's SRH is collected becomes important, since a person can experience several episodes of the common cold one year and only one or none the next year. On the other hand, chronic conditions are unlikely to vary much from year to year. To the best of our knowledge no study has ever tested this hypothesis. We therefore tested whether individuals are influenced by the frequency of their common cold episodes and we then tested the interaction effect between common cold and Charlson comorbidity on SRH.

## 2. Materials and Method

### 2.1. Study population

The present study used the 2005 Taiwan National Health Interview Survey (NHIS). The survey was conducted by the Bureau of Health Promotion in Taiwan (response rate = 80.6%). The survey consisted of 18,529 individuals > 18 years of age, of which 2727 individuals > 65 years of age). The subjects were selected using a multi-stage stratified systematic sampling. The target population for this survey were all individuals residing in Taiwan as identified by the National Registry Database. The sample was weighted so that it was representative of Taiwan's population at the year of the survey. Face-to-face interviews by well-trained interviewers were conducted using questionnaires.

To identify an individual's Charlson comorbidity score, the NHIS was linked to the 2004 claims data in the National Health Insurance Research Database (NHIRD). The NHIRD consists of all the individual records of inpatient and outpatient services utilization under the National Health Insurance (NHI). Taiwan's NHI is a public insurance system with compulsory enrollment by all citizens of Taiwan. Medical claims under the NHI were sent to the Bureau of National Health Insurance (BNHI) of Taiwan for cross-checking and validation to ensure the accuracy of the diagnosis coding.

Of the 18,529 subjects from the NHIS, 13,926 (75%) signed the consent for data linkage. It should be noted that there was a significant difference (*p* < 0.01) in terms of age and sex between those who agreed and those who disagreed to data linkage. Those who agreed tended to be younger and female. The generalizability of our results should be considered in light of this phenomenon. Of the 13,926 subjects, 13,723 (98.5%) had complete data on the variables used in this research, and were used as study subjects. All individual IDs were scrambled before the dataset was released to ensure individual information was being protected. This study was approved by the Institutional Review Board (IRB) of Yang-Ming University.

### 2.2. Measures

#### 2.2.1. Self-rated health

In the 2005 NHIS, people were asked to rate their general health by answering the question “How would you rate your general health status?” with the allowable responses including “Excellent”, “Very good”, “Good”, “Fair”, and “Poor”. These allowable responses were kept as the original 5 ordered categories in order to avoid any loss of information.

#### 2.2.2. Charlson comorbidity score

Part of the aim of our study was to test the effect of the Charlson comorbidity score on SRH. Thus it was imperative that the Charlson score was measured prior to the SRH being measured. Since the NHIS was carried out during various time periods during 2005, we calculated the Charlson comorbidity score over the period of January 1, 2004 to December 31, 2004, using the NHI claims data. The Charlson comorbidity score was calculated based on 17 disease categories. A person was considered having a comorbid condition in a year if s/he had at least 2 claim records with an International Classification of Disease, 9^th^ Revision (ICD-9) for that condition during the year. A higher score indicates a greater comorbidity.

#### 2.2.3. Number of common cold episodes

The number of common cold episodes during the period January 1, 2004 to December 31, 2004 was identified from the claims data based on the ICD-9 codes of 460-466 and 480-487. To avoid over-counting the number of episodes, if a person had more than 1 claim with the above ICD-9 codes within 14 days of the previous claim, then the incidence was treated as the same common cold episode.

#### 2.2.4. Other control variables

We also included many control variables, including the individual's sex, highest educational attainment and marital status. All these variables were obtained from the 2005 NHIS.

### 2.3. Statistical analysis

To ensure our results are comparable to previous studies, we used similar methodologies as proposed by Heller et al. [13] Table 1 shows the characteristics of the study sample by the SRH categories. An ordered logistic regression was estimated to determine the relationship between the Charlson comorbidity, the common cold and SRH (Table 2). The proportional odds assumption was evaluated and the assumption was met. The answers to the self-rated health question were the same as the original 5 categories, thus the odds ratios indicated a reporting of a higher ranked (that is, a worse) health status. Many studies convert continuous predictors by grouping values into two or more categories. This method of examining non-linear relationships often results in a loss of information.[21]. Multiple fractional polynomials offer a solution to this problem [22]. Here non-linearity in the continuous predictors is examined by fitting a first-order fractional polynomial. A best fitted power b_1_x*^p^* is compared with the linear model, where *p* comes from a set of candidates of −2, −1, −0.5, 0, 0.5, 1, 2, 3, and x^0^ denotes log x, and *p* = 1 means no transformation [22]. This set of powers offers considerable flexibility, and adding more powers usually does not significantly improve the model [22]. Model deviances are compared using a χ^2^ distribution with 1 degree of freedom. If hypothesis *p* = 1 is rejected then a non-linear function is preferred. Instead of just considering the eight first order polynomials, we also tested models with fractional polynomials of the second order (b_1_x*^p^* + b_2_x*^q^*) to increase model flexibility, with *p* and *q* chosen from the same candidates as described above. All continuous variables used in this study (including the number of common cold episodes and the age at baseline) were tested for all possible fractional polynomial models (8 first degree and 32 second degree models). These estimations were made using the *mfp* (multiple fractional polynomial) command in STATA MP. 12.0.

We also tested a number of interactions. More specifically, we tested the interaction between the Charlson comorbidity score and baseline age, common cold episodes and baseline age, and finally, Charlson's comorbidity and the number of common cold episodes. Charlson's comorbidity was fitted as a continuous variable in this case.

## 3. Results

Table 1 shows the baseline sample characteristics by SRH category. Most of the respondents rated their health as “Fair” (34.3%), followed by “Good” (30.6%) and “very good” (24.5%). The mean baseline age increases with a worse health rating. The mean Charlson score for the entire sample was 0.42. The Charlson score was highly skewed, as the number of subjects having a Charlson score equaling 0 was 79.7%.

Table 2 shows the ordered logistic regression estimated using fractional polynomials. For the continuous variables, only the second order term for common cold was significant.

Compared with those who had only primary school or less, those with a junior high school, senior high school, university or higher education had adjusted odds ratios of 0.78 (*p* = 0.001), 0.64 (*p* < 0.001) and 0.57 (*p* < 0.001) for reporting bad health, respectively. Those who were married were less likely to report bad health (OR = 0.79, *p* < 0.001) compared with those who were not married.

**Table 1.** Sample characteristics by levels of self-rated health in 2005.

	Total	Excellent		Very good		Good		Fair		Poor	
	n	n	%	n	%	n	%				
	13723	550		3,358		4,200		4,713		902	
Sex											
Female	6541	223	3.4	1439	22.0	1958	29.9	2,452	37.5	469.0	7.2
Male	7182	327	4.6	1919	26.7	2242	31.2	2,261	31.5	433.0	6.0
Baseline age (mean/sd)	42.79	37.69	14.8	39.08	14.5	41.16	15.2	44.97	16.7	55.83	18.7
25th percentile = 28.9											
50th percentile = 41.0											
75th percentile = 53.6											
Education											
Primary school or less	2434	64	2.6	369	15.2	599	24.6	1,100	45.2	302.0	12.4
Junior high school	1980	71	3.6	483	24.4	592	29.9	704	35.6	130.0	6.6
Senior high school	4734	218	4.6	1175	24.8	1475	31.2	1,562	33.0	304.0	6.4
University or higher	4575	197	4.3	1331	29.1	1534	33.5	1,347	29.4	166.0	3.6
Marital status											
Never married	4016	214	5.3	1130	28.1	1293	32.2	1,223	30.5	156.0	3.9
Married	8333	303	3.6	1982	23.8	2590	31.1	2,922	35.1	536.0	6.4
Divorced, widowed or other	1374	33	2.4	246	17.9	317	23.1	568	41.3	210.0	15.3
Charlson's comorbidity score in 2004 (mean/sd)	0.42	0.13	0.53	0.20	0.68	0.29	0.81	0.54	1.15	1.45	1.92
25th percentile = 0											
50th percentile = 0											
75th percentile = 0											
Number of episodes for common cold (mean/sd)	1.54	1.24	1.74	1.37	1.77	1.45	1.85	1.70	2.12	1.99	2.31
25th percentile = 0											
50th percentile = 1											
75th percentile = 2											

**Figure 1.**
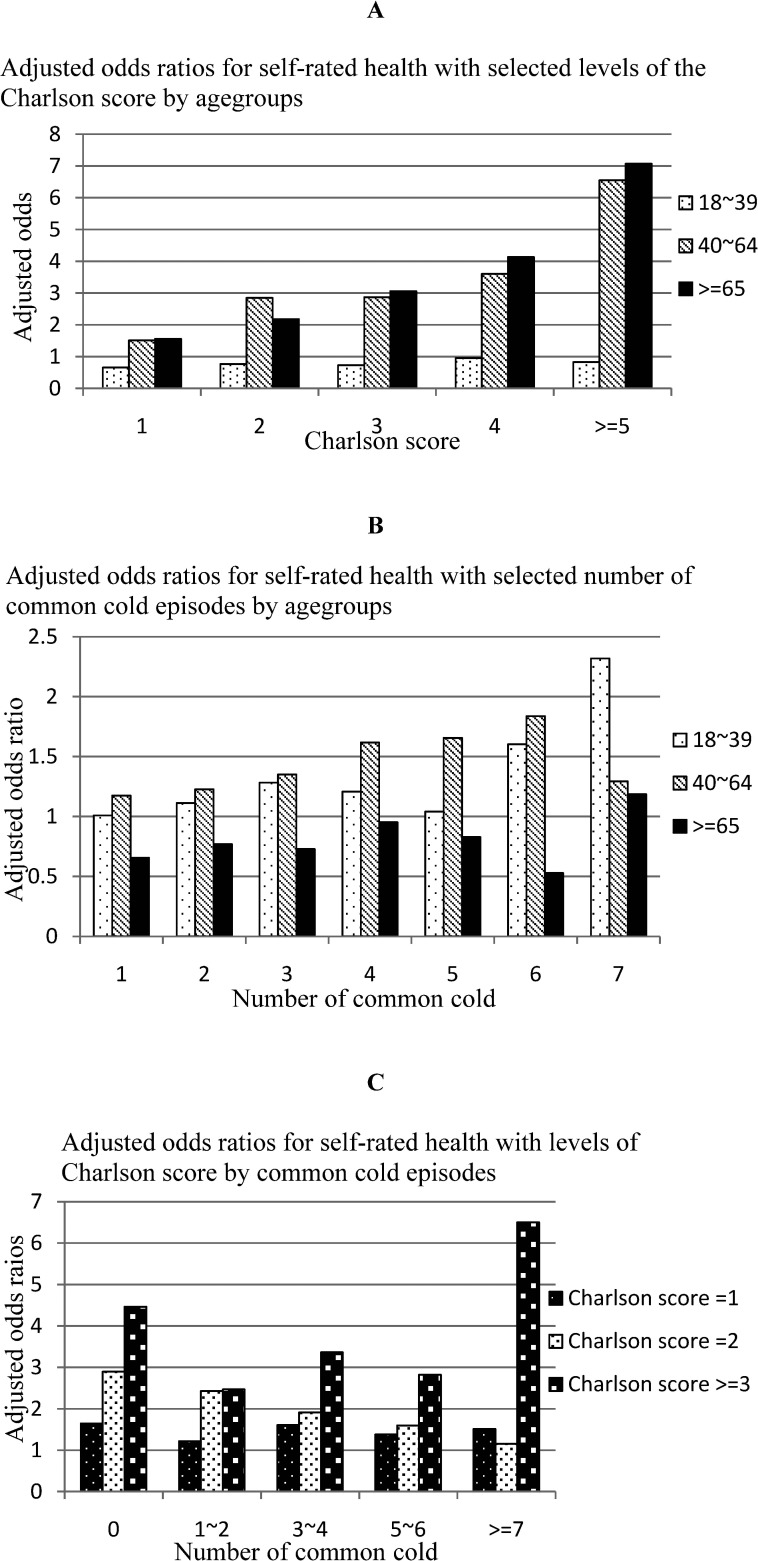
Adjusted odds ratios for selected levels of interaction by the Charlson comorbidity score, number of common cold episodes, and age groups.

Since the fractional polynomial model indicates a second-order fractional polynomial for the Charlson score, the number of common cold episodes and the baseline age, two terms will be created for each two-way interaction. Following Heller et al. [13], only the first-order function is used to create the interactions since it can avoid potential collinearity [13]. Figure 1 shows the adjusted odds ratios for different levels of the Charlson score and number of common cold episodes with the interaction effects. These odds ratios were estimated using a new model with the Charlson score fitted as a continuous variable. The reference group is the group with a Charlson score = 0. The interaction between the Charlson score and baseline age from the main model in Table 1 was significant. It is evident from Figure 1 (part A) that the effect of the Charlson score on reporting worse health differs significantly among the age groups. For the youngest age group (18∼39), a higher Charlson score did not lead to reporting worse health, and the adjusted odds ratios were less than 1 for all levels of the Charlson score. However, for the older age groups the situation was very different. For both the 40 ∼64 and >65 age groups, there was a graded association between a higher Charlson score and the likelihood of reporting bad health.

Part B of Figure 1 shows the adjusted odds ratios for the number of common cold episodes in reporting worse health by different age groups. The interaction term for these two variables was not statistically significant in the main model. Nevertheless, Figure 1 shows that the number of common cold episodes does not affect the oldest age group (≥ 65). In most cases, the odds ratios were less than 1.

Part C of Figure 1 shows that a higher Charlson score (Charlson score ≥ 3) has a larger effect on SRH for people with no common cold episodes, as well as for people with a very high frequency of common cold episodes (greater than 7). This bipolar effect is evident from the non-linear relationship between this interaction term and reporting bad health.

**Table 2.** Adjusted odds ratios (OR) from ordered logistic regression for reporting worsening health for the Charlson score and common cold episodes.

	SRH			
	Estimates	95% CI		*p*
Sex				
Female (reference)				
Male	1.405	1.271	1.555	< 0.001
Baseline age				
First order	1.020	0.960	1.084	0.515
Second order	0.995	0.965	1.025	0.729
Education				
Primary school (reference)				
Junior high school	0.778	0.670	0.902	0.001
Senior high school	0.643	0.561	0.737	< 0.001
University	0.573	0.498	0.660	<0.001
Marital status				
Never married				
Married	0.790	0.701	0.890	< 0.001
Divorced, widow or others	1.011	0.842	1.214	0.904
Charlson (2004)				
Myocardial infarction	1.457	0.526	4.041	0.469
Congestive heart failure	1.380	1.038	1.835	0.027
Peripheral vascular disease	1.869	1.067	3.275	0.029
Cerebrovascular disease	6.338	2.224	18.056	0.001
Dementia	2.170	0.933	5.047	0.072
Chronic pulmonary disease	1.163	0.851	1.591	0.343
Rheumatic disease	1.497	0.914	2.453	0.109
Peptic ulcer disease	1.677	1.359	2.069	< 0.001
Mild liver disease	1.515	1.235	1.860	< 0.001
Diabetes without chronic complication	3.332	1.735	6.398	< 0.001
Diabetes with chronic complication	2.023	1.265	3.236	0.003
Hemiplegia or paraplegia	2.146	1.736	2.653	< 0.001
Renal disease	2.171	1.446	3.260	< 0.001
Any malignancy,	40.917	6.109	274.042	< 0.001
Moderate or severe liver disease	0.692	0.258	1.858	0.465
Metastatic solid tumor	1.407	0.985	2.009	0.060
AIDS/HIV	2.182	0.806	5.907	0.125
Common cold				
First order	1.062	0.965	1.169	0.218
Second order	0.623	0.397	0.976	0.039

## 4. Discussion

This study examined whether or not individuals are influenced by the frequency of common cold episodes when asked to self-rate their health. Our study found that individuals do respond to the frequency of their common cold episodes. The fractional polynomial model indicates that both the Charlson score and the frequency of the common cold episodes have a non-linear relationship to SRH. Individuals suffering from the common cold at a lower frequency have a lower likelihood to report poor health than those with a higher frequency. The Charlson score and age has a significant interaction effect, which is consistent with the finding of Heller et al. [13]. In the present study we did not find a significant interaction between the common cold and age, although there was a trend indicating that the common cold has less of an effect on the elderly when it comes to reporting worsening health.

Recovery from a disease may be viewed as a positive component in health [1]. However, for short-lived health conditions such as common cold, a high frequency of occurrence still indicates poor health. To date, almost no studies have tested for changes in SRH over a short period of time. In most cases changes in SRH are measured using a period of one year [13],[23],[24]. Thus, the year at which the SRH information is collected becomes critical when taking the common cold into consideration, since the frequency of the common cold can differ substantially from year to year.

One possible explanation that people who experience a high frequency of the common cold have poor self-rated health is that people with a compromised immune system are more likely to experience a higher frequency of the common cold. A study found that the immune system may be associated with a persons' self-rated health [25]. It is also possible that people who have a poor immune system actually have poorer health and hence have poorer SRH.

It has been found that the older a person, the weaker the association between objective health and subjective health [26]. In the present study we found that this association depends on the type of objective condition. Older individuals tend not to take the common cold very much into consideration when rating their health. One explanation for this is that the elderly normally have a higher number of comorbidities, and they tend to consider them more serious than the short-lived common cold. On the other hand, the common cold may be the “main” health events for younger individuals if they are free of chronic conditions. Previous studies indicate that different factors have a different impact on different age groups. For example, one study [27] found that obesity has less of an impact on poor SRH for younger individuals compared with their older counterparts, even though it is well known that obesity is highly associated with chronic diseases [28].

We found a significant interaction between the Charlson score and the frequency of common cold episodes. A high Charlson score (≥ 3) had a higher impact on poor health rating when the individual's number of common cold episodes were either very low or very high. The reason behind this should be further investigated by future studies.

Our findings should be viewed in light of the study's strengths as well as its limitations. The strengths of this study are the use of fractional polynomial models, a nationally representative survey that includes all age groups, and the use of claims data rather than self-reported health conditions. However, there are some limitations to our study as follows. First, although the claims data are not subjected to recall bias, they nevertheless only measure the treated prevalence. In other words, if a person experiences the common cold but does not go to a medical institute for treatment, then this particular common cold episode will not have been captured. However, since Taiwan's NHI is compulsory for all citizens, and individuals only have to pay a minimal copayment for both the visit to the doctor and the medication prescribed, it is much cheaper for a person to visit a doctor, rather than stay home and pay any medication out of their own pocket. Consequently, the vast majority of common cold sufferers in Taiwan tend to see a physician for their common cold. Statistics have shown that the common cold accounts for most of the outpatient visits in Taiwan [29]. Thus the problem of underestimating the utilization of medical care for common cold episodes may be a moot point. It should also be noted that this study does not indicate any causal effect. There may be unmeasured confounders that explain the findings, such as for example, obesity, as mentioned above.

In conclusion, the present study indicates that individuals tend to respond to the frequency of their common cold episodes along with their chronic conditions when rating their health. Younger individuals tend to put more emphasis on the common cold than their older counterparts.
